# Students’ Socio-Emotional Skills and Academic Outcomes After the PROMEHS Program: A Longitudinal Study in Two European Countries

**DOI:** 10.3390/bs15111529

**Published:** 2025-11-10

**Authors:** Baiba Martinsone, Celeste Simões, Liberato Camilleri, Elisabetta Conte, Paula Lebre

**Affiliations:** 1 Department of Psychology, University of Latvia, LV-1586 Riga, Latvia; 2 Department of Education, Social Sciences and Humanities, Faculty of Human Kinetics, University of Lisbon, Cruz Quebrada, 1499-002 Lisbon, Portugal; csimoes@sapo.pt (C.S.); pmelo@fmh.ulisboa.pt (P.L.); 3 Institute of Environmental Health, Faculty of Medicine, University of Lisbon, Cruz Quebrada, 1649-028 Lisbon, Portugal; 4 Department of Statistics and Operations Research, University of Malta, MSD 2080 Msida, Malta; liberato.camilleri@um.edu.mt; 5 Department of Human and Social Sciences, University of Bergamo, 24129 Bergamo, Italy; elisabetta.conte@unibg.it; 6 Institute of Ethnomusicology—Center of Studies in Music and Dance (INET-md-FMH/ULisboa Branch), Cruz Quebrada, 1499-002 Lisbon, Portugal

**Keywords:** socio-emotional skills, academic outcome, PROMEHS program, longitudinal study, COVID-19 pandemic

## Abstract

Previous research shows that better socio-emotional skills are associated with students’ behavioral adjustment and positive learning outcomes; however, the protective role of socio-emotional skills regarding academic learning during global crises has not been studied sufficiently. This research aims to evaluate longitudinal changes in students’ socio-emotional skills and learning outcomes during the implementation of a universal prevention program Promoting Mental Health at Schools (PROMEHS). The research coincided with lockdown due to the COVID-19 pandemic, therefore allowing an assessment of the protective role of socio-emotional learning even in the face of adverse circumstances. In total, 3166 students (aged 7–17 years) from Latvia and Portugal participated in the research, and a survey of their respective teachers was used to collect data. The SSIS-SEL teacher form and a three-item learning outcomes measure addressing academic motivation, engagement, and performance were applied in the study. It was found that growth in socio-emotional skills has a positive effect on academic outcomes, while a decline in socio-emotional learning has a detrimental effect on academic outcomes. This applies to both experimental and control groups, both genders, all educational levels, and both countries. Additionally, the increase in socio-emotional skills predicted better academic outcomes in upper secondary schools than in primary and lower secondary schools.

## 1. Introduction

Schools, as one of the main life contexts for children and adolescents, are pivotal in their positive and healthy development. As such, schools should invest in their students’ holistic development and must focus not only on academic learning but also on social and emotional learning (European Commission, 2024a, 2024b; OECD, 2021, 2024; WHO, 2022). There is considerable evidence regarding schools’ role in students’ social and emotional learning and its positive impact on several related outcomes, including academic performance, across their academic trajectory (Cefai et al., 2021; Cipriano et al., 2023; DeSensi, 2024; Durlak et al., 2022; Lim et al., 2024).

Socio-emotional learning (SEL) is increasingly recognized as a fundamental and inseparable element of children’s and adolescents’ development, particularly in educational contexts. According to the Collaborative for Academic, Social, and Emotional Learning (CASEL, n.d.), SEL is the process through which children, adolescents, and adults acquire key social and emotional competencies to understand themselves and others. It is about building self-awareness and confidence to develop a strong and healthy sense of identity, learning to manage emotions in everyday life and challenging situations, and working toward personal and collective goals. Along the way, SEL helps people to develop empathy, form and maintain meaningful relationships, and make responsible decisions that support the well-being of themselves and those around them.

Social and emotional competencies are vital for positive development and significantly predict educational and professional achievement, health, and well-being (Schoon, 2021). Besides all the known positive outcomes of SEL, many social and emotional competencies are also considered key competencies for sustainability (Pacis & VanWynsberghe, 2020). SEL cultivates empathy, self-awareness, and critical thinking, empowering individuals to engage responsibly in creating a more sustainable and connected world.

The development and learning of social and emotional competencies are lifelong processes that begin in early childhood and continue into adulthood. These processes involve a dynamic interplay of physical, cognitive, emotional, and social factors since human development is inherently multidimensional (Lerner et al., 2015). As such, social and emotional development is deeply interconnected with cognitive and physical development, biological processes, social interactions, and cultural practices (Keller, 2020; Lamb, 2015).

Family is the first and main life context where SEL occurs through family interactions. The family plays a key role in this development by providing a safe and structured environment where children can learn to manage their emotions, cooperate with others, and internalize values and social norms (Grusec, 2011), allowing these competencies to be nurtured through consistent emotional support and guided social learning. These foundational experiences underscore SEL’s critical role in shaping children’s readiness to learn and succeed academically (e.g., Cipriano et al., 2023).

Schools are also uniquely positioned to foster SEL, serving as key contexts for holistic development. Eccles and Roeser (2015) conceptualize schools as multilevel developmental systems that influence students’ behavioral, emotional, and academic trajectories, highlighting their role in promoting ethical, civic, and socio-emotional development, besides the traditional focus on academic skills. Moreover, learning, besides being a cognitive process, is also a social and emotional one (Eccles & Roeser, 2015; Erickson & Cottingham, 2022; Pekrun, 2022). As Eccles and Roeser (2015) point out, teaching and learning are inherently and simultaneously social, emotional, cognitive, and moral processes because they involve the whole person. Moreover, Immordino-Yang et al. (2018) state that the provision of purposeful learning opportunities for young people requires teachers to attend to the development of the whole child in context. They also emphasize the need to involve the entire educational community through a whole-school approach in a collective effort to promote the health and well-being of all the community actors. This universal approach, addressing all children independently of their background, is in line with Weare (2015), who stated that the promotion of well-being and mental health through SEL in schools should not be considered a ‘luxury or optional extra’ but an evidence-based investment in the future of young people. Accordingly, SEL should be integrated into school life, not as an additional feature, but as a foundational component that brings gains in different developmental areas. Several studies revisited in systematic reviews and meta-analyses (Cipriano et al., 2023; Corcoran et al., 2018; Durlak et al., 2011, 2022; Goldberg et al., 2019; Lim et al., 2024; Taylor et al., 2017) have demonstrated that students engaged in well-designed and implemented SEL programs exhibited several positive outcomes, namely, gains in social and emotional competencies, a decrease in behavioral problems, higher levels of well-being, higher academic achievement, improved classroom behavior, better stress management, and increased attendance. Recent research indicates that better socio-emotional skills are associated with behavioral adjustment and positive learning outcomes even in preschool age (Conte et al., 2023; Martinsone et al., 2021). These findings indicate that SEL is not separate from the academically successful trajectory but rather a catalyst for it.

It has been emphasized that evidence-based programs are the preferred way to implement SEL, as they rely on science-based and age-appropriate approaches that gradually develop pupils’ socio-emotional skills while also proving effective in delivering positive changes in academic outcomes, behavioral adjustment, mental health, and citizenship (Greenberg, 2023). At the same time, such programs present challenges in terms of fidelity, ensuring that teachers in all classrooms and groups can implement SEL sustainably and precisely as the program is intended. The continuous professional development of educators and the provision of support to implementers require financial contributions. Thus, explicit SEL responds to these challenges by developing methodologies that support SEL indirectly or outside the framework of specific SEL programs. Typically, these are small routines that can be easily implemented in any subject lesson (Jones & Bouffard, 2012) or teaching or formative assessment strategy (Ferreira et al., 2020).

It is important not to oversimplify SEL as interventions that require little effort and investment do not sufficiently change an educator’s mindset and, therefore, practice. Oversimplified approaches to SEL programs that make them more attractive and easier to adopt could jeopardize their long-term sustainability (Commins & Elias, 1991). However, as schools must choose among different priorities daily, missing time is often found at the expense of classroom lessons, SEL lessons, or even separate activities fostering students’ socio-emotional growth. An issue, therefore, is creating a whole-school approach to building and sustaining SEL year by year, making it an integral part of the daily education process.

Aligned with this vast field of research are the European Union’s recommendations for this area. Social and emotional education in schools is a key element in the EU recommendations for promoting well-being and mental health at schools (Cefai et al., 2021; European Commission, 2024a, 2024b), and in turn, well-being is at the core of European education. According to the European vision, well-being is founded on opportunities for all, considering diversity, guaranteeing equity, and promoting inclusion (European Commission, 2024b). In recent years, several negative indicators regarding students’ mental health and well-being have worsened (OECD, 2016), which became more evident during the COVID-19 pandemic (Elharake et al., 2023; Naff et al., 2022). These aspects are recognized in the Council Recommendation on Pathways to School Success (Council of the European Union, 2022), particularly in the policy framework for school success, where the inclusion of SEL and mental health in curricula across the entire educational journey is included in the prevention measures. In addition, social and emotional competencies and mental health are viewed not merely as goals in themselves but as closely linked to academic success. Schools that prioritize a supportive environment and the establishment of positive and meaningful relationships, foster students’ socio-emotional competencies, address bullying and cyberbullying, support teachers and other staff well-being, and adopt a whole-school approach are more likely to see improvements in academic performance, reduced dropout rates, and overall healthier development in students (Council of the European Union, 2022).

The recent guidelines for school leaders, teachers, and educators (European Commission, 2024b) and education policymakers (European Commission, 2024a) also highlight the importance of the previously mentioned elements for successful learning. Within the 11 priorities established in these documents, it is possible to find the integration of social and emotional education in the school curriculum from the early years, along with promoting active student participation and empowerment, a positive climate, and safe schools. In addition, the prioritization of equity, inclusion, and diversity, the protection of fundamental rights, guaranteeing access to support services for students with mental health problems, teachers’ initial and continuous training, and strengthening the capacity of school leaders to promote well-being at schools, considering the challenges of the digital age, are vital components of an education system focused on successful holistic education and development.

Despite encouraging findings, significant gaps remain. First, the evidence base, which has expanded rapidly and internationally from contemporary meta-analytic syntheses, confirms broad though heterogeneous benefits for skills, attitudes, prosocial behavior, school climate/safety, and academic functioning (Cipriano et al., 2023). Results from multi-country evaluations in Europe indicate that universal programs can produce meaningful gains in students’ socio-emotional competence and prosocial behavior, alongside reductions in mental health difficulties (Cefai et al., 2022a).

Second, European longitudinal evidence is comparatively sparse and heterogeneous (e.g., Cipriano et al., 2023; Lim et al., 2024), indicating the need for context-sensitive, multi-year studies that track both SEL and academic trajectories and examine policy/implementation ecosystems (Cavioni et al., 2024). A Campbell-standard systematic review of Ibero-American studies (n = 22) reported the most consistent improvements in self-awareness, social awareness, self-control, relationship skills, decision-making, school climate, well-being, and academic achievement while highlighting gaps for sense of belonging and school safety. The results also pointed to the importance of dosage and curricular integration (Fernández-Martín et al., 2021). Technology-supported cooperative learning that embeds explicit SEL instruction has shown added value and scalability in Italian primary and middle schools (Zagni et al., 2025).

At the same time, population heterogeneity demonstrably matters. Latent-class analyses in Sweden identified distinct well-being trajectories under universal programming, implying that average effects obscure subgroup patterns (Sandell & Kimber, 2013). Program-specific trials echo this nuance. The Fast Track PATHS curriculum yielded modest but reliable multi-year reductions in aggression and increases in prosocial behavior and academic engagement, with stronger effects in less disadvantaged schools and for children with higher baseline aggression; several outcomes were moderated by gender (boys benefited more on peer-reported indices) (Bierman et al., 2010). The Second Step^®^ program improved classroom social climate when teachers delivered more lessons and used proactive classroom management (Osborne et al., 2025). In early childhood (ages 3–5), universal classroom programs produced moderate-to-large gains in social cooperation, interaction, and independence over a three-year period (Arráez et al., 2015). Moderator evidence from Making Choices similarly shows larger reductions in aggression and more positive social goals for boys than girls (Terzian et al., 2014). Longitudinal durability research shows uneven results, providing evidence of fadeout for both SEL and cognitive outcomes, as well as moderators tied to context, dosage, and implementation quality (Hart et al., 2024; West et al., 2020).

Such longitudinal evaluations offer a mixed view of how universal, school-based SEL effects endure over time. Evidence first points to sustained mental health benefits, for instance showing that school programs have maintained reductions in depressive symptoms over two to three years (Sato et al., 2012), and brief early interventions targeting anxiety and depression in preschoolers show lasting advantages, particularly for girls through middle adolescence (Rapee, 2013). In early childhood, gains in social competence, including cooperation, interaction, and independence, persist for more than two years in classroom and nursery contexts (Alba et al., 2015). By contrast, long-term effects on delinquency and substance use are less consistent: combined PATHS and Triple P evaluations report minimal durable impacts on these outcomes (Averdijk et al., 2016). At the same time, dosage and everyday exposure appear consequential. In a longitudinal follow-up on a Head Start RCT cohort, frequent (weekly to daily) third-grade exposure to universal SEL opportunities was associated with higher social skills, stronger student–teacher relationships, better academic skills, and lower impulsiveness (Zhai et al., 2015). Synthesizing across 86 RCTs with 56,662 participants, a meta-analysis indicates that both cognitive and socio-emotional impacts generally fade, with smaller post-test effects tending to persist more; at one- to two-year follow-up, conditional persistence was equal or greater for cognitive than for SEL outcomes, though small positive follow-up effects beyond what post-test impacts predict suggest some long-run benefits are under-captured at immediate post-test (Hart et al., 2024). Finally, developmental dynamics matter, as while academic achievement is relatively stable, SEL constructs such as self-efficacy, social awareness, and self-management are more context-sensitive and tend to decline after Grade 6, underscoring the need to time and adapt support during adolescence (West et al., 2020). Within the 7–17-year age range, developmental shifts require differentiated SEL design and assessment. In late childhood (approximately 7–11 years), children typically develop foundational skills. These include emotion labeling, simple self-regulation, and cooperative peer interaction. At this stage, SEL instruction often employs scaffolded routines and concrete activities that align with cognitive and social abilities. As students enter early adolescence (approximately 11–14 years), they experience biological, cognitive, and social re-orientation. Identity exploration, heightened peer belonging, and emerging abstract reasoning require more sophisticated SEL opportunities, such as self-reflection and peer conflict resolution. In later adolescence (approximately 14–17 years), SEL competencies become integrated into strategic goal-setting, civic reasoning, and sustained motivation. The developmental perspective within the CASEL framework argues that ignoring these age-specific transitions risks misalignment of goals, instruction, and assessment (e.g., CASEL, 2018). Effective SEL for 7–17-year-olds, therefore, requires both developmental sensitivity and ecological implementation.

Regarding academic impacts, research indicates that the effects vary by population and subject domain, with benefits appearing to be stronger when SEL is embedded in daily instruction, explicitly assessed, and aligned with equitable supports (Sert & Arıkan, 2025). Meta-analyses and international surveys suggest that SEL’s pathways to academics operate through improved emotion regulation, learning behaviors, and teacher–student relationships (Cipriano et al., 2023). Using OECD SSES data, open-mindedness and emotional regulation positively predicted mathematics achievement, with stronger associations for girls and students from lower-SES backgrounds; schools that formally assessed SEL also showed higher math scores (Sert & Arıkan, 2025). Despite this, not all trials find durable academic gains, as some detect short-term improvements that fade by later follow-ups (e.g., McCormick et al., 2021), whereas other long-term evidence indicates that socio-emotional skill improvements in childhood can drive educational attainment effects more than cognitive gains per se (Sorrenti et al., 2025).

In relation to longitudinal multi-informant evidence during COVID-19 (T1: October 2020; T2: May 2021) shows small increases in internalizing/externalizing difficulties and small decreases in SEL and prosocial behavior; importantly, adolescents with greater SEL development exhibited larger gains in resilience and prosociality and fewer difficulties over time (Martinsone et al., 2022a). Emerging causal evidence suggests that socio-emotional supports during remote learning mitigated losses in standardized math and Portuguese scores in Brazil (Lichand et al., 2024) and that Finnish students with stronger pre-pandemic SEL were better able to maintain or recover study engagement during higher-education transition (Mädamürk et al., 2025).

Studies in this area to date provide some evidence on the potential role of socio-emotional skills in promoting academic learning during unprecedented global disruptions (Levine et al., 2023; Quintiliani et al., 2021). Studies associate higher emotional intelligence and resilience with lower depression/anxiety/stress and better academic emotions and engagement during the pandemic (e.g., Levine et al., 2023; Malkoc et al., 2023; Barros & Sacau-Fontenla, 2021). Broader meta-analytic reviews argue that universal SEL can bolster coping with collective stressors and trauma through emotion regulation and resilience pathways (Durlak et al., 2022). Finally, systems-level analyses highlight that crises amplify inequities, eliciting that trauma-informed and culturally responsive SEL is essential for disadvantaged learners and refugee populations (e.g., Deitz et al., 2021).

Previous research has proven the role of socio-emotional competence in students’ academic adjustment (e.g., Greenberg, 2023). Nevertheless, the protective role of socio-emotional skills in academic learning during global crises like the COVID-19 pandemic still requires further analysis. Consequently, this paper aims to analyze longitudinal changes in students’ socio-emotional skills and academic outcomes after implementing the universal Promoting Mental Health at Schools (PROMEHS) program, which coincided with the lockdown due to the COVID-19 pandemic.

We hypothesize that growth in 7–17-year-old students’ socio-emotional skills between T1 (October 2020) and T2 (May 2021) has a positive relationship with academic outcomes, and this result applies to both experimental and control groups, both genders, all educational levels, and both countries. We also hypothesize that, despite the lockdown, the increase in SEL skills would have a more positive relationship with academic outcomes for the experimental group that received the PROMEHS program than the control group with no targeted intervention.

## 2. Materials and Methods

### 2.1. The Promoting Mental Health at Schools (PROMEHS) Program

The PROMEHS program was developed as part of the Erasmus+ Key Action 3 project “Promoting Mental Health at Schools,” which involved seven countries: Italy, Malta, Latvia, Croatia, Greece, Romania, and Portugal. The project aimed to design, implement, and assess a universal, school-based mental health curriculum for European students aged 3 to 18.

The theoretical framework of the curriculum is built on three key themes: (1) the promotion of SEL, (2) the promotion of resilience, and (3) the prevention of social, emotional, and behavioral problems. The first theme focuses on the five SEL competencies identified by CASEL (2018): self-awareness, self-management, social awareness, relationship skills, and responsible decision-making. The second theme relates to the ability to cope with psychosocial challenges (e.g., transitions, academic difficulties) and traumatic experiences (e.g., loss, chronic illness). The third theme concerns the prevention of internalizing problems (e.g., depression, anxiety, psychosomatic issues), externalizing problems (e.g., aggressive behavior), and at-risk behaviors, such as self-harm and addictions.

The PROMEHS program has several positive features (Conte et al., 2023). For instance, it is a universal intervention, meaning it is designed for the entire school community rather than only for clinical or at-risk populations. Furthermore, it adopts a whole-school approach, actively involving all key stakeholders in students’ school life (i.e., teachers, parents, school leaders, and policymakers). Each one is engaged in dedicated training designed to provide them with the knowledge and practical strategies needed to support children’s mental health both at school and at home. This collaborative effort ensures that all adults who play a significant role in children’s education consistently apply best practices to help students develop their skills and adopt positive behaviors across different contexts.

The program included teacher training and supervision, webinars for parents, and meetings with school leaders, all carried out by the PROMEHS developers, who have expertise in teacher training, school-based mental health promotion, and parenting. Trained teachers then implemented the PROMEHS curriculum with students in their classrooms. To ensure adherence to the planned activities, monitoring measures, such as checklists, rating scales, and questionnaires with both open- and closed-ended questions, were implemented before, during, and after the training sessions (Martinsone et al., 2022b).

As part of the program, teachers participated in a 16 h training course (online or in person) over one to two months. The training provided theoretical knowledge on both students’ and teachers’ mental health in educational settings. Topics included mental health statistics, the impact of the COVID-19 pandemic, stress and burnout, and strategies to enhance teachers’ mental health. Additionally, teachers were trained to use the PROMEHS handbooks, which contain practical activities (e.g., storytelling, self-reflection, group discussions, games, and role-playing) they could implement with their students in the classroom. The interactive nature of the training encouraged individual reflections and peer discussions.

After their training in October 2020, teachers implemented the PROMEHS curriculum in their classrooms over a 12-week period. Each week, they selected one activity from the handbook, preferably one that best addressed their students’ specific needs, aligned with academic content, and integrated into their lessons. The PROMEHS handbooks were differentiated according to students’ age. One handbook was suitable for preschool and primary school, while the other one was suitable for middle and secondary school. Within each handbook, every topic and competence was presented at both a basic and an advanced level, ensuring that activities were developmentally appropriate and adaptable to students’ age and abilities. Nevertheless, all PROMEHS activities followed the same structure. First, the teacher introduced a story from the handbook to address a specific ability (e.g., a story for primary school addressing social awareness encouraged students’ perspective-taking and empathy; for a complete list of topics and goals for the two handbooks, see Cefai et al., 2022b). After the story, teachers posed guiding questions to stimulate classroom discussion (e.g., “How do you think the character felt?”; “Have you ever experienced a similar situation? How did you react?”, etc.). This was followed by a practical task, carried out individually, in pairs, or in groups, such as creating a poster to display in the classroom or engaging in role-play to practice the targeted competence. Finally, each session concluded with a debriefing and reflection, during which the teacher explicitly highlighted the goal of the activity and linked it to students’ everyday experiences. Each activity lasted between one and two class lessons.

To ensure consistent and effective implementation, teachers received ongoing online supervision. Trainers organized three to four group supervision meetings for a total of nine hours (with approximately one session per month), where teachers shared their experiences, discussed the activities implemented in their classrooms, reflected on challenges, and described their observations on students’ progress. Monitoring tools, including checklists, self-reflection forms, and questionnaires, were used to systematically track teachers’ curriculum implementation.

To transfer and reinforce the newly acquired skills beyond the school environment, parents were encouraged to engage their children in complementary activities at home. Over the same 12-week period, teachers provided parents with a handbook containing activities that mirrored those conducted in the classroom. Home activities included parent–child discussions, games, and worksheets, requiring approximately 30 min to complete. To further engage families, parents participated in a series of three online webinars, each lasting approximately two hours. These sessions aimed to raise awareness of school mental health, support parents’ active role, promote reflection on good practices, and enhance the implementation of PROMEHS activities at home.

School leaders were also actively involved in the program. Trainers held six hours of individual meetings with each headteacher, either in person or remotely, constituting approximately one meeting per month. These meetings focused on school mental health policies, the impact of the COVID-19 pandemic, and effective strategies to create a positive school climate. Headteachers received a brief handbook on school mental health and were asked to reflect on ways to implement systemic improvements within their schools.

### 2.2. Participants

In total, 3166 students (aged 7–17 years) participated in the research, and this sample size guaranteed a maximum margin of error of 1.74%, assuming a 95% confidence level. Of these, 1722 students (922 in the experimental group and 800 in the control group) were from Latvia, and 1444 students (538 in the experimental group and 906 in the control group) were from Portugal. The students were stratified by both gender and age (see Table 1). The reports on students’ SEL and academic outcomes were received from their respective teachers.

### 2.3. Measures

*The Social Skills Improvement System Socio-Emotional Learning (SSIS SEL) Brief Scales* (Elliott et al., 2020). The teacher K-12 form was applied. The 20-item SSIS SEL Brief Scales measure the five domains of SEL: self-awareness, self-management, social awareness, relationship skills, and responsible decision-making. Each item was rated on a 4-point Likert scale, where 0 corresponds to ‘never’ and 3 corresponds to ‘almost always’. The SEL scale composite score, which was generated by averaging the rating scores of these 20 items, ranged from 0 to 3, where a larger score implies higher SEL competence.

*Academic outcome* was measured by considering three aspects: academic motivation, engagement in the learning process, and academic performance. These three aspects were measured on a 5-point Likert scale, where 0 corresponds to ‘very poor’ and 4 corresponds to ‘very good’. The academic outcome scale score, which was generated by averaging the rating scores of these three aspects, ranged from 0 to 4, where a higher score implies a better academic outcome. The three aspects were measured by asking teachers explicitly to rate students’ (1) academic motivation, (2) engagement in the learning process, and (3) academic performance. The three items were combined into a single scale since their pairwise correlations (0.846, 0.826, and 0.817) were positive and significantly larger than 0.

The explanatory variables included information provided by teachers about students’ nationality, age, gender, and school level.

Cronbach’s alpha was used to measure the internal consistency between the 20 SSIS SEL items describing aspects of SEL and between the three items measuring academic outcomes. This was carried out for each group (control, experimental) and phase (pre-test, post-test) combination. All Cronbach’s alphas (see Table 2) indicate that items of both measures have very good internal consistency to measure SEL and academic outcomes, and this applies for both groups and both phases.

### 2.4. Procedure

To evaluate changes in academic outcomes and socio-emotional competence, the students were stratified by group (experimental, control), gender (male, female), nationality (Latvia, Portugal), and school level (pre-primary, primary, lower secondary, upper secondary).

The pre-test (T1) and post-test (T2) time points were in October 2020 and May 2021, respectively. Each participant was assigned a unique code that was used to match students when comparing scores between the two phases. Only those questionnaires completed in both phases were included in the study.

In Latvia, researchers conducted informative campaigns across all schools in a particular region. Initially, agreements were established with school principals and teachers to secure their participation in the PROMEHS project. Informational letters, including informed consent forms, were sent to parents. Researchers later verified whether parental consent had been provided. Subsequently, researchers visited the schools and invited adolescents whose parents had given consent to participate in the PROMEHS project. Students also provided their own written informed consent. All surveys were administered in paper format, put into sealed envelopes, and collected by the researchers. Finally, researchers entered all responses into an electronic database. The Ethics Committee for Humanities and Social Sciences Research Involving Human Participants at the University of Latvia approved the research on 12 December 2019.

In Portugal, researchers first held meetings with local policymakers to present the PROMEHS project. These organizations then contacted school principals who were interested in participating in the study. In turn, each principal selected teachers willing to be involved in the project. Meetings were held with school principals, teachers, and school psychologists to introduce the project and explain the evaluation procedure. The researchers provided informed consent forms to teachers, who distributed them to parents. Teachers provided the requested information by using paper questionnaires or an online survey platform. When paper versions were used, teachers forwarded them to researchers, who then entered the data into the database. The Lisbon North Hospital Center and Lisbon Academic Medical Center Ethics Committee Faculty of Medicine, University of Lisbon, approved the research on 20 March 2020 (protocol code 74/20).

### 2.5. Analytic Approach

Two general linear models were fitted to investigate the impact of five explanatory variables on students’ academic outcomes and socio-emotional competence. The explanatory variables included group (experimental, control), time (pre-test, post-test), gender (male, female), nationality (Latvia, Portugal), and school level (pre-primary, primary, lower secondary, and upper secondary).

Correlation analysis was used to investigate the relationship between change in academic outcomes and change in socio-emotional competence. This was carried for students stratified by group, gender, nationality, and school level.

The change in socio-emotional competence score was generated by subtracting the socio-emotional competence score in the pre-test phase from the corresponding score in the post-test phase. The score ranges from −3 to 3, where a positive change implies improved SEL competence between the two phases and a negative change implies reduced SEL competence.

The change in academic outcome score was generated by subtracting the academic outcome score in the pre-test phase from the corresponding score in the post-test phase. The score ranges from −4 to 4, where a positive change implies an improved academic outcome between the two phases and a negative change implies a diminished academic outcome.

## 3. Results

### 3.1. General Linear Models

The goal of many research studies is to investigate collectively the impact of several explanatory variables on the dependent variable. It is well known that a single explanatory variable could be rendered a very important predictor in explaining variations in responses but unimportant in the presence of other explanatory variables. To address this issue, two general linear models were fitted to relate socio-emotional competence scores and academic outcome scores to five explanatory variables (time, country, group, student gender, and school level).

Table 3 and Table 4 show that time, group, student gender, and school level are significant predictors of socio-emotional competence scores; however, country had a negligible impact on the model fit. The parameter estimates show that, on average, the post-test score for socio-emotional competence was 0.080 scale points higher than the pre-test score. The experimental group scored 0.069 scale points more than the control group, and males scored 0.247 scale points less than females. Moreover, on average, the socio-emotional competence scores of pre-primary, primary, and lower secondary students were 0.083, 0.197, and 0.193 scale points higher, respectively, than those of upper secondary students.

Table 5 and Table 6 demonstrate that time, group membership, student gender, and school level were significant predictors of academic achievement, whereas country contributed minimally to model fit. Parameter estimates indicate that, on average, post-test scores were 0.093 scale points higher than pre-test scores. Students in the experimental group scored 0.065 scale points more than those in the control group, while male students scored 0.302 scale points less than female students. Additionally, pre-primary, primary, and lower secondary students outperformed upper secondary students by 0.494, 0.594, and 0.376 scale points, respectively.

### 3.2. Correlations Between Change in Socio-Emotional Competence and Change in Academic Outcomes

**Figure 1:**
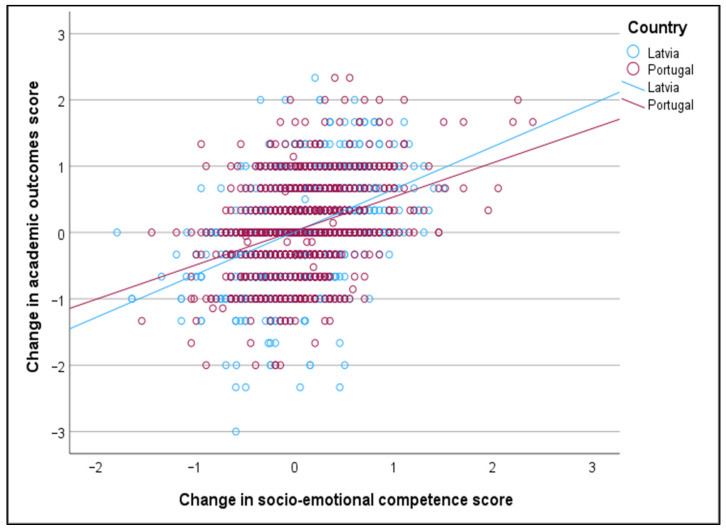
Relationship between change in socio-emotional competence and change in academic outcomes, stratified by country.

**Figure 2:**
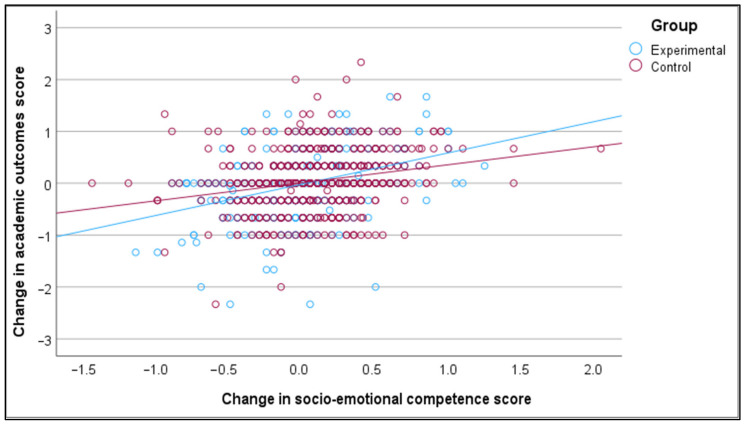
Relationship between change in socio-emotional competence and change in academic outcomes, stratified by group.

**Figure 3:**
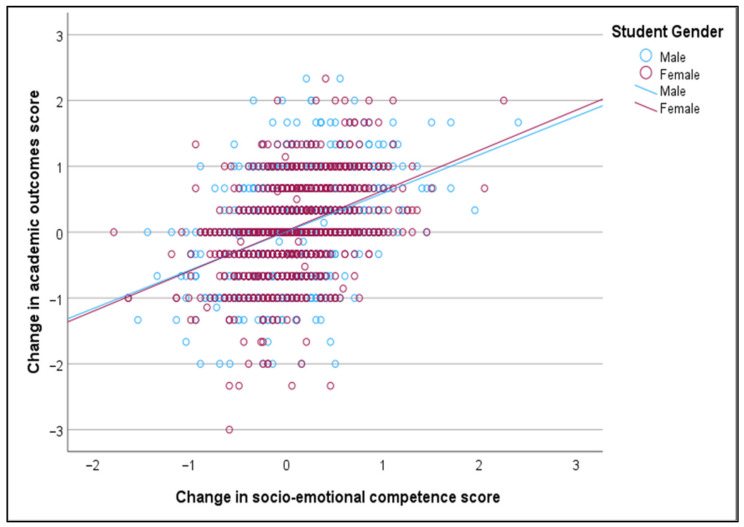
Relationship between change in socio-emotional competence and change in academic outcomes, stratified by gender.

**Figure 4:**
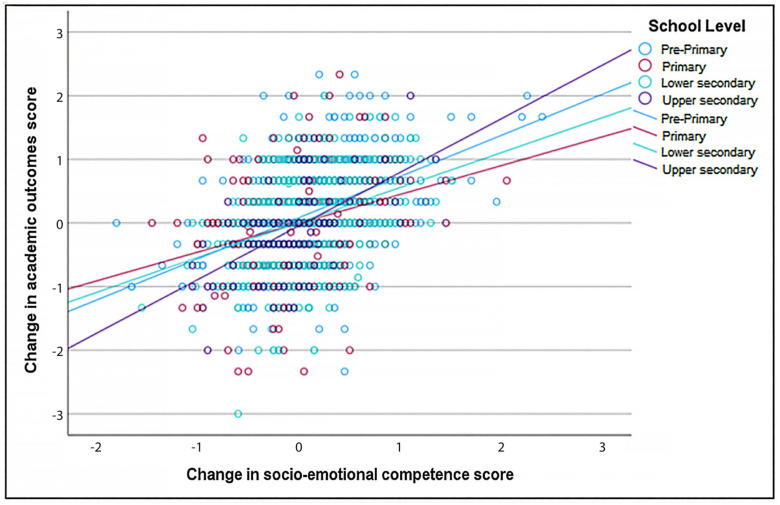
Relationship between change in socio-emotional competence and change in academic outcomes, stratified by education level.

The Pearson correlation coefficient shows that enhanced SEL competence has a statistically significant positive relationship with improved academic outcomes among 7–17-year-old students (r = 0.383, *p* < 0.001). Moreover, this positive and significant relationship is still valid when the sample is stratified by country (Latvia, Portugal), group (experimental, control), gender (male, female), and school level (pre-primary, primary, lower secondary, upper secondary) (see Table 7 and Figure 1, Figure 2, Figure 3 and Figure 4).

The positive relationship between the change in socio-emotional competence score and the change in academic outcome score is marginally stronger for Latvia compared to Portugal, marginally stronger for the experimental group compared to the control group, marginally stronger for females compared to males, and significantly stronger for upper secondary students compared to primary school students. The relationships between the change in socio-emotional competence score and the change in academic outcome score, stratified by country, group, student gender, and student education level, are displayed in Figure 1, Figure 2, Figure 3 and Figure 4.

The results confirm the hypothesis that growth in SEL competence has a significant positive relationship with enhanced academic outcomes, while a decline in SEL competence has a significant negative relationship with enhanced academic outcomes. This result applies to both experimental and control groups, both genders, all school levels, and both countries.

## 4. Discussion

A significant increment was found in both the socio-emotional competence and academic outcome scores between the pre- and post-test phases. These increments were more conspicuous for primary school female students in the experimental group compared to secondary school male students in the control group. Moreover, the two samples collected from Latvia and Portugal were fairly homogeneous, and mean scores varied marginally between the two countries. Consequently, the findings indicate that time, group assignment, gender, and school level are meaningful levers for improving socio-emotional competence, while country adds little explanatory power. The post-test gains and the experimental-over-control advantage align with the existing evidence that exposure to SEL programs and proper experimental implementation yield important gains in socio-emotional competencies (Cefai et al., 2021; Lim et al., 2024). The pronounced gender gap also aligns with previous literature, which indicates that girls tend to start their education with greater socio-emotional competence (e.g., empathy, emotion regulation, help-seeking) (Rudolph & Conley, 2005; Smith et al., 2019) and often gain more from school programs (Hajovsky et al., 2022). Several mechanisms have been highlighted in the literature regarding these differences. For one, girls more readily engage (Yang et al., 2018) with reflective, discussion-based activities common in SEL programs (Lin et al., 2022). Girls also seem to show a higher willingness to seek support from others (Sears & McAfee, 2017). Another aspect relates to the SEL program’s content, with many targeting internalizing challenges (e.g., stress, anxiety) (Cefai et al., 2021) that arise for girls during adolescence (Cosma et al., 2023), so the fit between developmentally appropriate needs and program content is tighter. Developmentally, the larger gains among pre-primary, primary, and lower secondary students compared to upper secondary students echo the view that earlier, sustained programs build skills more effectively than late efforts (Thomas & Aggleton, 2016). Additionally, upper secondary students face significant developmental tasks, such as social re-sorting, academic demands, and choosing professional paths, which can lead to a spike in stress (Torres & Mouraz, 2019; J. Wang et al., 2025). In these moments, targeted supports (mentoring, small-group SEL training, coping and stress-management modules) plus a positive school climate (supportive teacher–student relationships, clear norms, inclusive peer culture) can amplify gains and narrow risk trajectories (Cefai et al., 2021). The negligible contribution of the country to the model fit suggests that universal processes and school-level conditions (e.g., how programs are delivered in classrooms, how students develop socially, and how schools are organized) may play a leading role (Roffey, 2017; Vroom et al., 2020).

Regarding academic achievement, the pattern of effects (post-test gains, experimental-over-control gains, sizable gender differences, and more solid outcomes for younger students compared to older students) suggests that exposure, developmental stage, and gender are the most actionable factors for improving academic achievement through SEL. The impact of socio-emotional learning on academic outcomes has been well documented in several systematic reviews and meta-analyses (e.g., Corcoran et al., 2018; Durlak et al., 2022). Nevertheless, current evidence regarding the gender differences observed in this study is scarce. Most studies do not disaggregate results by gender, and the available evidence suggests similar overall benefits (Correia & Marques-Pinto, 2016), or a more substantial impact on academic outcomes for girls (Sert & Arıkan, 2025). Qualitative research indicates that marginalized boys, particularly from diverse racial backgrounds, may face unique challenges in emotional expression and engagement, highlighting the need for culturally responsive SEL interventions (Koltz et al., 2025; Wilson, 2025). Bearing these results in mind, improving classroom emotional support and organizing learning environments are key mechanisms linking SEL participation to academic gains for all students of all ages (McCormick et al., 2015).

Regarding grade differences, the results only showed a positive impact of the PROMEHS program’s implementation on academic outcomes for primary school students. Previous research has shown mixed results, ranging from no significant differences between school levels (Shi & Cheung, 2024; Shi et al., 2025) to differences depending on the academic subject (Corcoran et al., 2018). On the other hand, some studies indicate a greater impact of SEL programs on younger students (e.g., Cook et al., 2018; McCormick et al., 2021; Park & Blair, 2020; Simões et al., 2021). Several factors may explain why SEL programs tend to have a greater impact on academic outcomes in younger (i.e., elementary school) students. Firstly, younger students are at a critical developmental stage during which their brains have more plasticity (Low et al., 2019; Osawa & Konishi, 2003; Mira-Galvañ & Gilar-Corbi, 2020), making it easier for them to integrate and apply SEL skills to leverage academic outcomes (Cook et al., 2018). Being the only teacher of the class can be another contributing factor since elementary school teachers have more opportunities to integrate SEL into daily routines and classroom activities, creating a consistent and supportive learning environment (Low et al., 2019; Mira-Galvañ & Gilar-Corbi, 2020). Parents’ involvement can also play a role. Research shows that parental involvement has a consistently positive relationship with academic achievement in elementary school children (Erdem & Kaya, 2020; Wilder, 2014), but in middle school, this relationship is less consistent (Otani, 2020; Patall et al., 2008), and it tends to diminish in high school (Cui et al., 2023). Despite the challenges posed by the COVID-19 pandemic, some studies show that parents devoted more time to educational activities with their children, including teaching, facilitating, and providing resources (Coniglio et al., 2025). Moreover, research indicates that the pandemic fostered greater collaboration between parents and teachers, leading to improved home-school communication and understanding, supporting children’s education during remote learning (Gola et al., 2024; Levy, 2023). This may also have contributed to the results.

Regarding the PROMEHS program’s outcomes, it was expected that an increase in SEL skills would have a more positive effect on academic outcomes for the experimental group than the control group. When considering the whole sample of 7–17-year-olds, the rate of change in the academic outcome score relative to the SEL score was greater for the experimental groups compared to the control groups; however, the difference was not statistically significant. We could speculate that the 12-week intervention was not long enough to see significant changes between the groups in terms of academic outcomes. Furthermore, it is possible that academic outcomes during the pandemic were not considered relevant (e.g., compared to people’s health and well-being), so the scores provided by teachers may have been underestimated. The wide age range of the whole sample should also be taken into account. While implementing the PROMEHS program, significant differences emerged across age groups due to the COVID-19 pandemic. Younger students continued in-person schooling to some extent, whereas older students participated in fully remote learning and attended PROMEHS sessions online.

Despite evidence of the effectiveness of the PROMEHS program in showing gains in socio-emotional competence and prosocial behavior and a decrease in mental health issues (externalizing and internalizing problems) (Cefai et al., 2022a), no significant changes were observed in academic outcomes across the whole sample of six participating European countries. Several possible explanations have been advanced, including the duration and dosage of the program, its implementation in the pandemic context, and the measurement instrument used for academic outcomes (Cefai et al., 2022b). Nevertheless, in some countries, such as Italy (Conte et al., 2023, for pre-primary students) and Portugal (Simões et al., in press, for the whole sample), an increase in academic achievement was observed in the experimental group, which aligns with several studies that highlight the positive impact of SEL programs on academic outcomes (Cipriano et al., 2023; Corcoran et al., 2018; Durlak et al., 2011, 2022; Shi et al., 2025).

It was found that improvement in socio-emotional skills across time had a positive relationship with the academic outcomes of students aged 7–17 years, despite the wide age range of the research group. This result applied to both experimental and control groups, male and female students, all educational levels, and both countries. This result points to the universal nature of this finding, as evidenced by the results of other studies (e.g., Cipriano et al., 2023; Corcoran et al., 2018; Durlak et al., 2011, 2022; Lim et al., 2024).

According to Huang and Zeng (2023), the relationship between socio-emotional skills and academic performance is often mediated by factors such as self-efficacy and teacher-student relationships. Self-efficacy enhances students’ confidence in their abilities and influences how they approach challenges, manage stress, adapt, and persist in academic tasks (Castro Torres et al., 2023; Cruz et al., 2025). The impact of caring teacher-student relationships on academic outcomes is very well known (Austin et al., 2013; Pianta & Stuhlman, 2004; Velasquez et al., 2013). Caring practices within schools promote student engagement by fostering an affinity for learning, helping students maintain emotional stability, and succeeding in academic work (Austin et al., 2013). A recent review indicates that positive teacher-student relationships, in addition to academic engagement, have a significant role in academic achievement; conversely, a negative teacher-student relationship potentially aggravates disengagement and underachievement, especially in vulnerable groups (Di Lisio et al., 2025).

Notably, this significant relationship between increases in socio-emotional skills and academic outcomes was also valid during the situation of adversity when schools provided remote learning due to the COVID-19 pandemic. Other studies have found similar results. For instance, Y. Wang et al. (2023) found that emotional competence and online learning readiness were positively associated with academic results during the pandemic. According to the authors, high emotional competence supported students’ resilience when facing COVID-19-related challenges, helping them to learn effectively online. Similarly, Lichand et al. (2024), reporting an intervention during the pandemic, showed that socio-emotional skills mitigated the adverse effects of remote learning. The intervention, targeting students’ socio-emotional skills, was associated with increased standardized test scores relative to the control group, preventing 7.5% of math and 24% of Portuguese learning losses.

Interestingly, when students were grouped by school level, the increase in socio-emotional skills predicted better academic outcomes in upper secondary schools than in primary and lower secondary schools. Considering our results, we could speculate that older students with better SEL skills were better able to implement self-regulated learning during the lockdown. Some studies seem to align with this hypothesis. The development of older students’ cognitive and socio-emotional skills, resulting in more complex and integrated capabilities, allows them to use these skills more effectively than younger students for academic success (Davis et al., 2014; Hachem et al., 2022; Tze et al., 2022). Other studies highlight specific skills, like self-efficacy, indicating that, as students grow older, their belief in their capabilities to achieve academically becomes a strong predictor of academic outcomes (Cruz et al., 2025), or a growth mindset, that becomes more critical as students face more challenges related to the educational environments and transitions and that, in turn, help them to cope and succeed facing these challenges (Svensen, 2025). Another hypothesis is that factors such as mental health and school connectedness could significantly mediate the relationship between socio-emotional skills and academic outcomes in older adolescents (Panayiotou et al., 2019). Although the present study was conducted during the COVID-19 pandemic, and this argument seems misaligned with the mental problems and isolation associated with the lockdown measures imposed on the population, the ability to manage distress, which is more developed in older students (Davis et al., 2014), may explain the results of the present study, giving an advantage to older students and making them more resilient to this adversity.

## 5. Conclusions

It was found that enhanced socio-emotional competence has a positive relationship with academic outcomes even during the strong restrictions in educational settings necessitated by the COVID-19 pandemic. Moreover, this positive and significant relationship remains valid when the sample is stratified by country (Latvia, Portugal), group (experimental, control), gender (male, female), or school level (pre-primary school, primary, lower secondary, upper secondary). It was also found that growth in SEL has a positive relationship with enhanced academic outcomes, while a decline in SEL has a negative relationship with enhanced academic outcomes. The PROMEHS program’s implementation had an effect on academic outcomes for primary school students, particularly girls, whereas the increase in socio-emotional competence predicted a greater increase in academic outcomes among upper-secondary students.

This research has several limitations. The first is its reliance exclusively on teachers’ evaluations of students’ academic performance and outcomes, which may introduce bias due to subjective perceptions. Additionally, the quasi-experimental design without random assignment may have limited the researchers’ ability to establish causal inferences between participation in the PROMEHS program and changes in students’ academic and socio-emotional competencies. The strong positive relationship between change in socio-emotional competence and change in academic outcome does not imply causation since it is difficult to identify which of the two variables is the cause and which is the effect.

Additional challenges included the variability in school closures, remote learning arrangements, and the variability in how the program was delivered due to challenges for schools and teachers to maintain regular sessions (online or in person) caused by the changing regulations during the pandemic. The well-being of families should also be taken into account with regard to their ability to support their children’s academic success during the pandemic. Also, the remote learning that was necessary during the lockdown period may have jeopardized teachers’ ability to evaluate their students’ academic outcomes. Finally, future research is recommended to examine differences across different age groups and educational stages in greater depth to identify specific intervention needs at both the practical and conceptual levels.

## Figures and Tables

**Table 1:** Socio-demographic characteristics of study participants (n = 3166).

Variable	Categories	Frequency	Percentage
Country	Latvia	1722	54.4%
	Portugal	1444	45.6%
Group	Experimental	1460	46.1%
	Control	1706	53.9%
School Level	Pre-primary	808	25.5%
	Primary	1199	37.9%
	Lower Secondary	1047	33.1%
	Higher Secondary	112	3.4%
Student Gender	Male	1557	49.2%
	Female	1609	50.8%

**Table 2:** Cronbach’s alpha measuring internal consistency between the items.

Scale	Group and Phase	Cronbach’s Alpha	Number of Items
Socio-emotional competence	Experimental, Pre-test	0.947	20
	Experimental, Post-test	0.952	20
	Control, Pre-test	0.941	20
	Control, Post-test	0.944	20
Academic outcome	Experimental, Pre-test	0.946	3
	Experimental, Post-test	0.957	3
	Control, Pre-test	0.936	3
	Control, Post-test	0.945	3

**Table 3:** Tests of between-subjects effects (dependent variable: socio-emotional competence).

Predictor	Sum of Squares	df	Mean Square	F	*p*-Value
Intercept	25,077.667	1	25,077.667	10,0479.9	<0.001
Time	10.058	1	10.058	40.300	<0.001
Country	0.480	1	0.480	1.923	0.166
Group	6.810	1	6.810	27.287	<0.001
Student Gender	95.681	1	95.681	383.370	<0.001
School Level	20.510	3	6.837	27.392	<0.001
Error	1569.103	6287	0.250		

**Table 4:** General linear model (dependent variable: socio-emotional competence).

Predictor Category	Par. Est.	Std. Error	t	*p*-Value
Intercept	3.199	0.036	90.105	<0.001
Time (Post-Test)	0.080	0.013	6.348	<0.001
Time (Pre-Test)	0			
Country = Latvia	−0.018	0.013	−1.387	0.166
Country = Portugal	0			
Group = Experimental	0.069	0.013	5.224	<0.001
Group = Control	0			
Student Gender = Male	−0.247	0.013	−19.580	<0.001
Student Gender = Female	0			
School Level = Pre-Primary	0.083	0.037	2.249	0.025
School Level = Primary	0.197	0.036	5.446	<0.001
School Level = Lower Secondary	0.193	0.036	5.320	<0.001
School Level = Upper Secondary	0			

**Table 5:** Tests of between-subjects effects (dependent variable: academic outcomes).

Predictor	Sum of Squares	df	Mean Square	F	*p*-Value
Intercept	35,200.475	1	35,200.475	45,192.758	<0.001
Time	6.729	1	6.729	8.639	0.003
Country	1.084	1	1.084	1.392	0.238
Group	12.490	1	12.490	16.035	<0.001
Student Gender	142.930	1	142.930	183.503	<0.001
School Level	101.103	3	33.701	43.268	<0.001
Error	4909.384	6303	0.779		

**Table 6:** General linear model (dependent variable: academic outcomes).

Predictor Category	Par. Est.	Std. Error	t	*p*-Value
Intercept	3.468	0.063	55.308	<0.001
Time (Post-Test)	0.093	0.023	4.004	<0.001
Time (Pre-Test)	0			
Country = Latvia	−0.027	0.023	−1.180	0.238
Country = Portugal	0			
Group = Experimental	0.065	0.022	2.939	0.003
Group = Control	0			
Student Gender = Male	−0.302	0.022	−13.546	<0.001
Student Gender = Female	0			
School Level = Pre-Primary	0.494	0.065	7.558	<0.001
School Level = Primary	0.594	0.064	9.311	<0.001
School Level = Lower Secondary	0.376	0.064	5.869	<0.001
School Level = Upper Secondary	0			

**Table 7:** Pearson correlations between growth in socio-emotional competence and academic outcomes stratified by country, group, gender, and school level.

Grouping Variable	Category	Pearson Correlation
Country	Latvia	0.410 ***
	Portugal	0.351 ***
Group	Experimental	0.393 ***
	Control	0.372 ***
Student Gender	Male	0.379 ***
	Female	0.387 ***
School Level	Pre-primary	0.434 ***
	Primary	0.298 ***
	Lower Secondary	0.356 ***
	Upper Secondary	0.565 ***

*** *p* < 0.001.

## Data Availability

The raw data supporting the conclusions of this article will be made available by the authors without undue reservation.

## References

[B1-behavsci-15-01529] Alba G., Fernández-Cabezas M., Justicia F., Pichardo M. C. (2015). The longitudinal effect of the Aprender a Convivir (learning to live together) programme in childhood: The development of social competence [Efecto longitudinal del programa Aprender a Convivir en la infancia: Desarrollo de la competencia social]. Cultura y Educación.

[B2-behavsci-15-01529] Arráez A., Pichardo M. C., Justicia F. (2015). Longitudinal study of the effects of the aprender a convivir program on children’s social competence [Estudio longitudinal de los efectos del programa Aprender a Convivir en la competencia social infantil]. Revista de Psicodidactica/Journal of Psychodidactics.

[B3-behavsci-15-01529] Austin G., Bates S., Duerr M. (2013). Guidebook to the California healthy kids survey.

[B4-behavsci-15-01529] Averdijk M., Zirk-Sadowski J., Ribeaud D., Eisner M. (2016). Long-term effects of two childhood psychosocial interventions on adolescent delinquency, substance use, and antisocial behavior: A cluster randomized controlled trial. Journal of Experimental Criminology.

[B5-behavsci-15-01529] Barros C., Sacau-Fontenla A. (2021). New insights on the mediating role of emotional intelligence and social support on university students’ mental health during COVID-19 pandemic: Gender matters. International Journal of Environmental Research and Public Health.

[B6-behavsci-15-01529] Bierman K. L., Coie J. D., Dodge K. A., Greenberg M. T., Lochman J. E., McMahon R. J., Pinderhughes E. (2010). The effects of a multiyear universal social–emotional learning program: The role of student and school characteristics. Journal of Consulting and Clinical Psychology.

[B7-behavsci-15-01529] CASEL (n.d.). What is social and emotional learning?.

[B8-behavsci-15-01529] CASEL (2018). Keeping SEL developmental: The importance of a developmental lens for fostering and assessing SEL competencies.

[B9-behavsci-15-01529] Castro Torres M. E., Vargas-Piérola P. M., Pinto C. F., Alvarado R. (2023). Multiple sequential mediation model of the effect of social capital investment on academic stress. International Journal of Educational Research Open.

[B10-behavsci-15-01529] Cavioni V., Broli L., Grazzani I. (2024). Bridging the SEL CASEL framework with European educational policies and assessment approaches. International Journal of Emotional Education.

[B11-behavsci-15-01529] Cefai C., Camilleri L., Bartolo P., Grazzani I., Cavioni V., Conte E., Ornaghi V., Agliati A., Gandellini S., Tatalovic Vorkapic S., Poulou M., Martinsone B., Stokenberga I., Simões C., Santos M., Colomeischi A. A. (2022a). The effectiveness of a school-based, universal mental health programme in six European countries. Frontiers in Psychology.

[B12-behavsci-15-01529] Cefai C., Camilleri L., Bartolo P., Grazzani I., Cavioni V., Conte E., Ornaghi V., Agliati A., Gandellini S., Tatalović Vorkapić S., Poulou M., Martinsone B., Stokenberga I., Simões C., Santos M., Colomeischi A. A. (2022b). Promoting mental health in school. Evaluating the effectiveness of the PROMEHS Programme in improving students’ and teachers’ social and emotional competence, resilience and mental health.

[B13-behavsci-15-01529] Cefai C., Simões C., Caravita S. (2021). A systemic, whole-school approach to mental health and well-being in schools in the EU. NESET Analytical report..

[B14-behavsci-15-01529] Cipriano C., Strambler M. J., Naples L. H., Ha C., Kirk M., Wood M., Sehgal K., Zieher A. K., Eveleigh A., McCarthy M., Funaro M., Ponnock A., Chow J. C., Durlak J. (2023). The state of evidence for social and emotional learning: A contemporary meta-analysis of universal school-based SEL interventions. Child Development.

[B15-behavsci-15-01529] Commins W. W., Elias M. J. (1991). Institutionalization of mental health programs in organizational contexts: The case of elementary schools. Journal of Community Psychology.

[B16-behavsci-15-01529] Coniglio N. D., Hoxhaj R., Jayet H. (2025). Coping with education supply shocks: How COVID-19 affected parents’ time spent on children’s education. Applied Economics Letters.

[B17-behavsci-15-01529] Conte E., Cavioni V., Ornaghi V., Agliati A., Gandellini S., Santos M. F., Santos A. C., Simões C., Grazzani I. (2023). Supporting preschoolers’ mental health and academic learning through the PROMEHS program: A training study. Children.

[B18-behavsci-15-01529] Cook C. R., Low S., Buntain-Ricklefs J., Whitaker K., Pullmann M. D., Lally J. (2018). Evaluation of second step on early elementary students’ academic outcomes: A randomized controlled trial. School Psychology Quarterly.

[B19-behavsci-15-01529] Corcoran R. P., Cheung A. C., Kim E., Xie C. (2018). Effective universal school-based social and emotional learning programs for improving academic achievement: A systematic review and meta-analysis of 50 years of research. Educational Research Review.

[B20-behavsci-15-01529] Correia K., Marques-Pinto A. (2016). «Giant Leap 1»: A social and emotional learning program’s effects on the transition to first grade. Children and Youth Services Review.

[B21-behavsci-15-01529] Cosma A., Abdrakhmanova S., Taut D., Schrijvers K., Catunda C., Schnohr C. (2023). A Focus on adolescent mental health and well-being in Europe, Central Asia and Canada: Health behaviour in school-aged children international report from the 2021/2022 survey.

[B22-behavsci-15-01529] Council of the European Union (2022). Council recommendation of 28 November 2022 on pathways to school success and replacing the council recommendation of 28 June 2011 on policies to reduce early school leaving. Official Journal of the European Union.

[B23-behavsci-15-01529] Cruz A. M., Carlo G., Gulseven Z., Vandell D. L. (2025). A longitudinal study of prosocial behaviors predicting later academic performance in US Latine early adolescents. Journal of Adolescence.

[B24-behavsci-15-01529] Cui T., Wang C., Liu Q., Liu J. (2023). Domains of parental involvement and academic achievement across grade levels in the Chinese context. Asia Pacific Journal of Education.

[B25-behavsci-15-01529] Davis A., Solberg V. S., de Baca C., Gore T. H. (2014). Use of social emotional learning skills to predict future academic success and progress toward graduation. Journal of Education for Students Placed at Risk.

[B26-behavsci-15-01529] Deitz R., Lahmann H., Thompson T. (2021). Social and emotional learning (SEL) systematic review.

[B27-behavsci-15-01529] DeSensi V. L. (2024). Embedding social and emotional (SEL) design to support college student well-being and learning outcomes: A review of the relevant literature. Journal of the National Organization for Student Success.

[B28-behavsci-15-01529] Di Lisio G., Milá Roa A., Halty A., Berástegui A., Couso Losada A., Pitillas C. (2025). Nurturing bonds that empower learning: A systematic review of the significance of teacher-student relationship in education. Frontiers in Education.

[B29-behavsci-15-01529] Durlak J. A., Mahoney J. L., Boyle A. E. (2022). What we know, and what we need to find out about universal, school-based social and emotional learning programs for children and adolescents: A review of meta-analyses and directions for future research. Psychological Bulletin.

[B30-behavsci-15-01529] Durlak J. A., Weissberg R. P., Dymnicki A. B., Taylor R. D., Schellinger K. B. (2011). The impact of enhancing students’ social and emotional learning: A meta-analysis of school-based universal interventions. Child Development.

[B31-behavsci-15-01529] Eccles J. S., Roeser R. W., Bornstein M. H., Lamb M. E. (2015). School and community influences on human development. Developmental science: An advanced textbook.

[B32-behavsci-15-01529] Elharake J. A., Akbar F., Malik A. A., Gilliam W., Omer S. B. (2023). Mental health impact of COVID-19 among children and college students: A systematic review. Child Psychiatry and Human Development.

[B33-behavsci-15-01529] Elliott S. N., DiPerna J. C., Anthony C. J., Lei P. W., Gresham F. M. (2020). SSIS SEL Brief Scales—Teacher K-12.

[B34-behavsci-15-01529] Erdem C., Kaya M. (2020). A meta-analysis of the effect of parental involvement on students’ academic achievement. Journal of Learning for Development.

[B35-behavsci-15-01529] Erickson R. J., Cottingham M. D., Daniel D., Samson A. C., Walle E. A. (2022). Emotion development in context. The Oxford handbook of emotional development.

[B36-behavsci-15-01529] European Commission (2024a). Wellbeing and mental health at school—Guidelines for education policymakers.

[B37-behavsci-15-01529] European Commission (2024b). Wellbeing and mental health at school—Guidelines for school leaders, teachers and educators.

[B38-behavsci-15-01529] Fernández-Martín F.-D., Romero-Rodríguez J.-M., Marín-Marín J.-A., Gómez-García G. (2021). Social and emotional learning in the Ibero-American context: A systematic review. Frontiers in Psychology.

[B39-behavsci-15-01529] Ferreira M., Martinsone B., Talic S. (2020). Promoting sustainable social emotional learning in schools through relationship-centered learning environment, teaching methods and formative assessment. Journal of Teacher Education for Sustainability.

[B40-behavsci-15-01529] Gola G., Calvo S., Castelli L., Egloff M., Negrini L., Piatti A., Rocca L., Sahlfeld W. (2024). Communication between pupils, parents and teachers: Research on school during the COVID-19 pandemic. The encyclopedia of COVID.

[B41-behavsci-15-01529] Goldberg J. M., Sklad M., Elfrink T. R., Schreurs K. M. G., Bohlmeijer E. T., Clarke A. M. (2019). Effectiveness of interventions adopting a whole school approach to enhancing social and emotional development: A meta-analysis. European Journal of Psychology of Education.

[B42-behavsci-15-01529] Greenberg M. T. (2023). Evidence for social and emotional learning in schools.

[B43-behavsci-15-01529] Grusec J. E. (2011). Socialization processes in the family: Social and emotional development. Annual Review of Psychology.

[B44-behavsci-15-01529] Hachem M., Gorgun G., Chu M.-W., Bulut O. (2022). Social and emotional variables as predictors of students’ perceived cognitive competence and academic performance. Canadian Journal of School Psychology.

[B45-behavsci-15-01529] Hajovsky D. B., Caemmerer J. M., Mason B. A. (2022). Gender differences in children’s social skills growth trajectories. Applied Developmental Science.

[B46-behavsci-15-01529] Hart E. R., Bailey D. H., Luo S., Sengupta P., Watts T. W. (2024). Fadeout and persistence of intervention impacts on social–emotional and cognitive skills in children and adolescents: A meta-analytic review of randomized controlled trials. Psychological Bulletin.

[B47-behavsci-15-01529] Huang C., Zeng X. (2023). Social and emotional development of disadvantaged students and its relationship with academic performance: Evidence from China. Frontiers in Psychology.

[B48-behavsci-15-01529] Immordino-Yang M. H., Darling-Hammond L., Krone C. (2018). The brain basis for integrated social, emotional and academic development: How emotions and social relationships drive learning.

[B49-behavsci-15-01529] Jones S. M., Bouffard S. M. (2012). Social and emotional learning in schools: From programs to strategies. Social Policy Report.

[B50-behavsci-15-01529] Keller H. (2020). Children’s socioemotional development across cultures. Annual Review of Developmental Psychology.

[B51-behavsci-15-01529] Koltz J. L., Deliman A., Daines E. (2025). Section 4: Case studies and best practices in SEL: Transforming schools with SEL: Success stories from marginalized communities. The power of social and emotional learning for student success.

[B52-behavsci-15-01529] Lamb M. E., Lamb M. E., Lerner R. M. (2015). Processes underlying social, emotional, and personality development: A preliminary survey of the terrain. Handbook of child psychology and developmental science, vol. 3: Socioemotional processes.

[B53-behavsci-15-01529] Lerner R. M., Hershberg R. M., Hilliard L. J., Johnson S. K., Bornstein M. H., Lamb M. E. (2015). Concepts and theories of human development. Developmental science: An advanced textbook.

[B54-behavsci-15-01529] Levine R. S., Lim R. J., Bintliff A. V. (2023). Social and emotional learning during pandemic-related remote and hybrid instruction: Teacher strategies in response to trauma. Education Sciences.

[B55-behavsci-15-01529] Levy R. (2023). Home-school communication: What we have learned from the pandemic. Education 3–13.

[B56-behavsci-15-01529] Lichand G., Christen J., Egeraat E. V. (2024). Neglecting students’ socio-emotional skills magnified learning losses during the pandemic. npj Science of Learning.

[B57-behavsci-15-01529] Lim J. H., Rho E., Yang C. (2024). Evidence-based practices of culturally responsive social and emotional learning (SEL) programs: A systematic review and meta-analysis. School Psychology Review.

[B58-behavsci-15-01529] Lin T.-J., Kraatz E., Ha S. Y., Hsieh M.-Y., Glassman M., Nagpal M., Sallade R., Shin S. (2022). Shaping classroom social experiences through collaborative small-group discussions. British Journal of Educational Psychology.

[B59-behavsci-15-01529] Low S., Smolkowski K., Cook C., Desfosses D. (2019). Two-year impact of a universal social-emotional learning curriculum: Group differences from developmentally sensitive trends over time. Developmental Psychology.

[B60-behavsci-15-01529] Malkoc S., Macher D., Hasenhütl S., Paechter M. (2023). Good performance in difficult times? Threat and challenge as contributors to achievement emotions and academic performance during the COVID-19 outbreak. Frontiers in Psychology.

[B61-behavsci-15-01529] Martinsone B., Stokenberga I., Damberga I., Supe I., Simões C., Lebre P., Canha L., Santos M., Santos A. C., Fonseca A. M., Santos D., Gaspar de Matos M., Conte E., Agliati A., Cavioni V., Gandellini S., Grazzani I., Ornaghi V., Camilleri L. (2022a). Adolescent social emotional skills, resilience and behavioral problems during the COVID-19 pandemic: A longitudinal study in three European countries. Frontiers in Psychiatry.

[B62-behavsci-15-01529] Martinsone B., Stokenberga I., Grazzani I. (2022b). Monitoring system of implementation of the Promoting Mental Health at Schools (PROMEHS) program. Frontiers in Psychology.

[B63-behavsci-15-01529] Martinsone B., Supe I., Stokenberga I., Damberga I., Cefai C., Camilleri L., Bartolo P., O’Riordan M. R., Grazzani I. (2021). Social emotional competence, learning outcomes, emotional and behavioral difficulties of preschool children: Parent and teacher evaluations. Frontiers in Psychology.

[B64-behavsci-15-01529] Mädamürk K., Upadyaya K., Hietajärvi L., Lonka K., Salmela-Aro K. (2025). The importance of socio-emotional skills obtained before the COVID-19 pandemic in supporting study engagement during the pandemic and transition to higher education. European Journal of Psychology of Education.

[B65-behavsci-15-01529] McCormick M. P., Cappella E., O’Connor E. E., McClowry S. G. (2015). Social-emotional learning and academic achievement: Using causal methods to explore classroom-level mechanisms. AERA Open.

[B66-behavsci-15-01529] McCormick M. P., Neuhaus R., O’Connor E. E., White H. I., Horn E. P., Harding S., Cappella E., McClowry S. (2021). Long-term effects of social-emotional learning on academic skills: Evidence from a randomized trial of INSIGHTS. Journal of Research on Educational Effectiveness.

[B67-behavsci-15-01529] Mira-Galvañ M.-J., Gilar-Corbi R. (2020). Design, Implementation and evaluation of an emotional education program: Effects on academic performance. Frontiers in Psychology.

[B68-behavsci-15-01529] Naff D., Williams S., Furman-Darby J., Yeung M. (2022). The mental health impacts of COVID-19 on PK–12 students: A systematic review of emerging literature. AERA Open.

[B69-behavsci-15-01529] OECD (2016). Trends shaping education 2016.

[B70-behavsci-15-01529] OECD (2021). Beyond academic learning: First results from the survey of social and emotional skills.

[B71-behavsci-15-01529] OECD (2024). Social and emotional skills for better lives: Findings from the OECD survey on social and emotional skills 2023.

[B72-behavsci-15-01529] Osawa M., Konishi Y. (2003). Developing the brain—Proposal to child neurologists: How to nurture and stimulate brain development. No to Hattatsu = Brain and Development.

[B73-behavsci-15-01529] Osborne K. R. M., Low S., Smolkowski K. (2025). Benefits to classroom social climates following two years of SEL program implementation: A Second Step^®^ evaluation. School Psychology Review.

[B74-behavsci-15-01529] Otani M. (2020). Parental involvement and academic achievement among elementary and middle school students. Asia Pacific Education Review.

[B75-behavsci-15-01529] Pacis M., VanWynsberghe R. (2020). Key sustainability competencies for education for sustainability: Creating a living, learning and adaptive tool for widespread use. International Journal of Sustainability in Higher Education.

[B76-behavsci-15-01529] Panayiotou M., Humphrey N., Wigelsworth M. (2019). An empirical basis for linking social and emotional learning to academic performance. Contemporary Educational Psychology.

[B77-behavsci-15-01529] Park E.-Y., Blair K.-S. C. (2020). Check-in/check-out implementation in schools: A meta-analysis of group design studies. Education and Treatment of Children.

[B78-behavsci-15-01529] Patall E. A., Cooper H., Robinson J. C. (2008). Parent involvement in homework: A research synthesis. Review of Educational Research.

[B79-behavsci-15-01529] Pekrun R., Daniel D., Samson A. C., Walle E. A. (2022). Development of achievement emotions. The Oxford handbook of emotional development.

[B80-behavsci-15-01529] Pianta R. C., Stuhlman M. W. (2004). Teacher-child Relationships and children’s success in the first years of school. School Psychology Review.

[B81-behavsci-15-01529] Quintiliani L., Sisto A., Vicinanza F., Curcio G., Tambone V. (2021). Resilience and psychological impact on Italian university students during COVID-19 pandemic. Distance learning and health. Psychology, Health & Medicine.

[B82-behavsci-15-01529] Rapee R. M. (2013). The preventative effects of a brief, early intervention for preschool-aged children at risk for internalising: Follow-up into middle adolescence. Journal of Child Psychology and Psychiatry.

[B83-behavsci-15-01529] Roffey S. (2017). The ASPIRE principles and pedagogy for the implementation of social and emotional learning and the development of whole school well-being. International Journal of Emotional Education.

[B84-behavsci-15-01529] Rudolph K. D., Conley C. S. (2005). The socioemotional costs and benefits of social-evaluative concerns: Do girls care too much?. Journal of Personality.

[B85-behavsci-15-01529] Sandell R., Kimber B. (2013). Heterogeneity in responses to a universal prevention program. The Journal of Primary Prevention.

[B86-behavsci-15-01529] Sato S., Ishikawa S., Togasaki Y., Ogata A., Sato Y. (2012). Long-term effects of a universal prevention program for depression in children: A 3-year follow-up study. Child and Adolescent Mental Health.

[B87-behavsci-15-01529] Schoon I. (2021). Towards an integrative taxonomy of social-emotional competences. Frontiers in Psychology.

[B88-behavsci-15-01529] Sears H. A., McAfee S. M. (2017). Seeking help from a female friend: Girls’ competencies, friendship features, and intentions. Personal Relationships.

[B89-behavsci-15-01529] Sert M. A., Arıkan S. (2025). Investigating the differential relationship between the big five domains of social and emotional skills and mathematics achievement. International Electronic Journal of Elementary Education.

[B90-behavsci-15-01529] Shi J., Cheung A. C. (2024). Effective components of social emotional learning programs: A meta-analysis. Journal of Youth and Adolescence.

[B91-behavsci-15-01529] Shi J., Cheung A. C., Ni A. (2025). A meta-analysis of effective social-emotional learning programs in Pre-K-12 classrooms: Disentangling the critical role of curriculum-based approaches in promoting students’ social emotional skills. Current Psychology.

[B92-behavsci-15-01529] Simões C., Santos A. C., Lebre P., Daniel J. R., Branquinho C., Gaspar T., Matos M. G. d. (2021). Assessing the impact of the European resilience curriculum in preschool, early and late primary school children. School Psychology International.

[B93-behavsci-15-01529] Simões C., Santos M., Lebre P., Canha L., Santos A. C., Fonseca A. M., Santos D., Murgo C., Mato M. G., Grazzani I. (in press). Promover a Saúde mental nas escolas—Impacto do currículo europeu PROMEHS em Portugal.

[B94-behavsci-15-01529] Smith S., Dutcher K., Askar M., Talwar V., Bosacki S. (2019). Emotional competencies in emerging adolescence: Relations between teacher ratings and student self-reports. International Journal of Adolescence and Youth.

[B95-behavsci-15-01529] Sorrenti G., Zölitz U., Ribeaud D., Eisner M. (2025). The Causal Impact of Socio-Emotional Skills Training on Educational Success. Review of Economic Studies.

[B96-behavsci-15-01529] Svensen E. (2025). Growth mindset and academic achievement: A multilevel analysis of upper secondary school completion. Scandinavian Journal of Educational Research.

[B97-behavsci-15-01529] Taylor R. D., Oberle E., Durlak J. A., Weissberg R. P. (2017). Promoting positive youth development through school-based social and emotional learning interventions: A meta-analysis of follow-up effects. Child Development.

[B98-behavsci-15-01529] Terzian M. A., Li J., Fraser M. W., Day S. H., Rose R. A. (2014). Social information-processing skills and aggression. Research on Social Work Practice.

[B99-behavsci-15-01529] Thomas F., Aggleton P. (2016). A confluence of evidence: What lies behind a “whole school” approach to health education in schools?. Health Education.

[B100-behavsci-15-01529] Torres A. C., Mouraz A. N. A. (2019). Transition to upper secondary education in Portugal: Students’ voices about academic difficulties. Educacao e Sociedade.

[B101-behavsci-15-01529] Tze V. M. C., Li J. C.-H., Daniels L. M. (2022). Similarities and differences in social and emotional profiles among students in Canada, USA, China, and Singapore: PISA 2015. Research Papers in Education.

[B102-behavsci-15-01529] Velasquez A., West R., Graham C., Osguthorpe R. (2013). Developing caring relationships in schools: A review of the research on caring and nurturing pedagogies. Review of Education.

[B103-behavsci-15-01529] Vroom E. B., Massey O. T., Yampolskaya S., Levin B. L. (2020). The impact of implementation fidelity on student outcomes in the Life Skills Training Program. School Mental Health.

[B104-behavsci-15-01529] Wang J., Kaufman T., Mastrotheodoros S., Branje S. (2025). Longitudinal associations between academic motivation and school-related stressors in adolescents transitioning to secondary school. Learning and Instruction.

[B105-behavsci-15-01529] Wang Y., Xia M., Guo W., Xu F., Zhao Y. (2023). Academic performance under COVID-19: The role of online learning readiness and emotional competence. Current Psychology.

[B106-behavsci-15-01529] Weare K. (2015). What works in promoting social and emotional well-being and responding to mental health problems in schools?.

[B107-behavsci-15-01529] West M. R., Pier L., Fricke H., Hough H., Loeb S., Meyer R. H., Rice A. B. (2020). Trends in student social-emotional learning: Evidence from the first large-scale panel student survey. Educational Evaluation and Policy Analysis.

[B108-behavsci-15-01529] WHO (2022). World mental health report: Transforming mental health for all.

[B109-behavsci-15-01529] Wilder S. (2014). Effects of parental involvement on academic achievement: A meta-synthesis. Educational Review.

[B110-behavsci-15-01529] Wilson K. (2025). Section 3: Implementing SEL in schools and policy implications: Breaking barriers to SEL: Advancing equity and access in underserved communities. The power of social and emotional learning for student success.

[B111-behavsci-15-01529] Yang C., Bear G. G., May H. (2018). Multilevel associations between school-wide social-emotional learning approach and student engagement across elementary, middle, and high schools. School Psychology Review.

[B112-behavsci-15-01529] Zagni B., Van Ryzin M., Ianes D., Scrimin S. (2025). Advancing social and emotional skills through tech-supported cooperative learning in primary and middle schools. European Journal of Education.

[B113-behavsci-15-01529] Zhai F., Raver C. C., Jones S. M. (2015). Social and emotional learning services and child outcomes in third grade: Evidence from a cohort of Head Start participants. Children and Youth Services Review.

